# Gene variants of *CYP1A1* and *CYP2D6* and the risk of childhood acute lymphoblastic leukaemia; outcome of a case control study from Kashmir, India

**Published:** 2017-06

**Authors:** Sadiq Nida, Bhat Javid, Masood Akbar, Shah Idrees, Wani Adil, Ganai Bashir Ahmad

**Affiliations:** 1 Department of Biochemistry, University of Kashmir, Hazratbal Srinagar, Jammu and Kashmir, India; 2 Department of Clinical Heamatology, Sher-e-Kashmir Institute of Medical Sciences (SKIMS), Srinagar, Jammu and Kashmir, India; 3 Center of Research Development (CORD), University of Kashmir Srinagar, Jammu and Kashmir, India

**Keywords:** Acute Lymphoblastic Leukaemia, Polymorphism, Kashmir, Xenobiotics

## Abstract

Studies on associations of various polymorphisms in xenobiotic metabolizing genes with different cancers including acute lymphoblastic leukaemia (ALL) are mixed and inconclusive. The current study analyzed the relationship between polymorphisms of phase I xenobiotic metabolizing enzymes, cytochromes P450 1A1 (*CYP1A1*) and *CYP2D6* and childhood ALL in Kashmir, India. We recruited 200 confirmed ALL cases, and an equal number of controls, matched for sex, age and district of residence to the respective case. Information was obtained on various lifestyle and environmental factors in face to face interviews with the parents/attendants of each subject. Genotypes of *CYP1A1* and *CYP2D6* were analyzed by polymerase chain reaction and restriction fragment length polymorphism method. Logistic regression models were used to calculate odds ratios (ORs) and 95% confidence intervals (95% CIs). Compared to the GG genotype, we found a higher ALL risk in subjects who harbored variant (AA) genotype (OR=20.9; 95% CI: 6.01-73.1, P<0.0001) and AG genotype (OR=42.6; 95% CI: 8.3-217.5, P<0.0001) of CYP2D6*4 polymorphism. Although, we found a significant association of CYP1A1*2A polymorphism with ALL risk, but the risk did not persist in the adjusted model (OR=6.76; 95% CI: 0.63–71.8, P=0.100). The study indicates that unlike CYP1A1*2A, CYP2D6*4 polymorphism is associated with ALL risk. However, more replicative studies with larger sample size are needed to substantiate our findings.

## INTRODUCTION

Genetic susceptibility and environmental exposures play roles in the etiology of leukemia [1]. Environmental exposures like ionizing radiation, benzene, and cytotoxic therapy are some of the proposed causes of acute leukemia and for genetic susceptibility, single nucleotide polymorphism (SNP) the most common type that consists of a variation at a single base pair. Depending on where it is located, SNPs can interfere with a gene’s function, affecting metabolic pathways and thus affecting the course of the disease and its progress. SNPs in the xenobiotic system, cell regulation, and DNA repair system have been identified as risk factors in childhood leukemia [2, 3]. Therefore, functional polymorphisms in genes encoding carcinogen-metabolizing enzymes may have relevance in determining susceptibility to pediatric cancer [4].

All xenobiotics, including therapeutic agents, are metabolized and eliminated from the body by a system of enzymes encoded by specific genes. Most of these genes are polymorphic and some polymorphic forms have an altered enzyme activity [5]. As the cytochrome group represents the first line of defense against toxic chemicals, carcinogens and used drugs [6], the genetic variants in these xenobiotic metabolizing enzymes can significantly affect the susceptibility to childhood acute lymphoblastic leukemia (ALL) [7]. For this reason, two genes of this cytochrome family *CYP1A1* and *CYP2D6* have gained much interest and many genetic variants have been reported in both the genes.

Kashmir valley located at a high altitude in the northern part of India, have preserved genetic pool mostly due to consanguineous marriages [8]. Owing to this factor and its geographical location Kashmiris show wide genetic diversity from the rest of India. There is no population-based tumor registry at this moment and the various studies relating to the epidemiologic facts of cancer in Kashmir are essentially hospital based. As per the recent study conducted by Muzaffar et al., [9] on pattern and profile of childhood malignancies in Kashmir showed that ALL is the leading childhood malignancy which accounts for ~37% of the total childhood malignant cases and reportedly child is exposed to a range of xenobiotics through maternal and paternal lifestyle habits. But the role of polymorphisms in genes involved in such xenobiotic metabolism, the interaction among them and with the environment is not yet studied in Kashmir. Therefore we conducted a case-control study in Kashmir to assess the risk of ALL associated with polymorphisms in *CYP1A1* and *CYP2D6*.

## MATERIALS AND METHODS

**Study subjects and data collection : **This study included 200 newly diagnosed histopathological confirmed childhood ALL patients and 200 controls. ALL patients were diagnosed as per French–American-British (FAB) criteria [10, 11] in the Division of Clinical Haematology of Sher-i-Kashmir Institute of Medical Sciences (SKIMS), only tertiary care hospital in the whole Kashmir Valley located in Srinagar, the central city in Kashmir valley. This study was conducted between March 2012 and December 2015. The inclusion criteria for ALL cases were (1) Complete clinical history was available; (2) Patients below the age of 20 years (3) Subjects of Kashmir origin. All the controls were recruited from SKIMS and the criteria for inclusion in the control group were: (1) Kashmiri patients enrolled for minor ailments (like a hernia, urinary stones, diarrhoea, appendicitis, prostatitis, pancreatitis, fever workup, jaundice, biliary stones, trauma/accidents, infections, and fractures). (2) Age, gender, and district matched with respective ALL cases and (3) had no history of any malignancy. The research protocol was approved by the Institutional Ethics Committee of SKIMS and informed consent was obtained from all participating individuals or parents involved in the study. Structured questionnaires were used to collect information on age, sex, place of residence, parental education, smoking; family history, monthly income and other possible confounding factors of interest in face to face interviews. No proxies were used in the study.

**Collection of blood sample and genotyping** **: **Two milliliters of venous blood was collected from each patient in EDTA coated plastic vial and stored at −80ºC before DNA extraction. Genomic DNA was extracted from blood samples by using the phenol-chloroform method [12]. The DNA extracted was quantified and stored at -20ºC until used for polymerase chain reaction (PCR) and restriction fragment length polymorphism (RFLP). The CYP1A1*2A (rs4646903) and CYP2D6*4 (**rs3892097**) polymorphisms were determined as described previously [13, 14]. 

**Statistical** **Analysis: **Categorical variables were set for presenting and calculating numbers and percentages for different variants of *CYP2D6* and *CYP1A1*. Conditional logistic regression models were used to calculate odds ratios (ORs) and corresponding 95 % confidence intervals (CIs) to assess the association of various polymorphisms of CYP2D6*4 and CYP1A1*2A with childhood ALL risk and to assess the possible gene–gene and gene–environment interactions. The adjustment was made for known risk factors like age, sex, residence, parental education level, monthly income, parental occupation, smoking, family history of cancer and in utero X-ray exposure during pregnancy. All statistical analysis was done using Stata software, version 12 (STATA Corp., College Station, TX, USA). Two-sided P<0.05 was considered as statistically significant.

## RESULTS

Distribution of demographic factors, the wealth scores a socioeconomic indicator, paternal smoking, and allele frequency by case status are shown in Table 1. The majority of cases were ≤5 years of age and 60% were males. A number of ALL cases resided in rural areas than respective controls. Distribution of non-genetic factors in cases and controls including paternal smoking status around the period of conception (P=0.045), paternal occupation (P<0.001) and in utero X-ray exposure during pregnancy (P=0.045) was significantly different among cases and controls. However, no significant differences were observed between parental education (P=0.317), monthly income (P=0.230) and family history of cancer (P=0.527) among cases and controls. 

Genotypic frequencies of both *CYP1A1* and *CYP2D6* in ALL cases and controls are summarized in Table 2. We found that both variant (CC) and heterozygous genotype (CT) of CYP1A1*2A polymorphism were associated with ALL risk, but this risk did not persist in the adjusted model (OR=6.76, P>0.100), when results were adjusted for potential confounders. Further, the association did not persist when CC and TT genotypes were grouped together in the adjusted model (OR=1.36, P=0.002). 

**Table 1:** Demographic characters of childhood ALL cases and controls

**Variables**		**Cases n (%)**	**Controls n (%)**	** P** a
**Age**	≤5	110 (55.0)	110 (55.0)	1.000
	6-10	60 (30.0)	60 (30.0)	
	>10	30 (15.0)	30 (15.0)	
				
**Gender**	Male	120 (60.0)	120 (60.0)	1.000
	Female	80 (40.0)	80 (40.0)	
				
**Dwelling**	Urban	65 (32.5)	65 (32.5)	1.000
	Rural	135 (67.5)	135 (67.5)	
				
**Paternal occupation**	Govt. employee	20 (10.0)	55 (27.5)	<0.001
	Business	28 (14.0)	45 (22.5)	
	Farmer	66 (33.0)	54 (27.0)	
	Labour	86 (43.0)	46 (23.0)	
				
**Paternal smoking**	Yes	115 (57.5)	95 (47.5)	0.045
	No	85 (42.5)	105 (52.5)	
				
**Monthly income**	<10,000	108 (54.0)	96 (48.0)	0.230
	>10000	92 (46.0)	104 (52.0)	
				
**Parental education**	Yes	95 (47.5)	105 (52.5)	0.317
	No	105 (52.5)	95 (47.5)	
				
**In utero X-ray exposure during pregnancy**	Yes	105 (52.5)	85 (42.5)	0.045
	No	95 (47.5)	115 (57.5)	
				
**Family history of cancer **	Yes	72 (36.0)	66 (33.0)	0.527
	No	128 (64.0)	134 (67.0)	

a Chi- square test (χ2) was used to calculate *P*-values for categorical variables. *n*, number of individuals

**Table 2:** Distribution of CYP1A1*2A and CYP2D6*4 genotypes among cases and controls and their interaction among themselves in modulating the risk of ALL in Kashmir, India

**Variable**	**Cases n (%)**	**Controls n (%)**	**Crude OR** 1 **(95% CI)**^2^	**Adj OR** 3 ** (95% CI)** 2	**P **
***CYP1A1***					
TT	142 (71.0)	168 (84.0)	1.0	1.0	-
CT	53 (26.5)	31 (15.5)	2.14 (1.30 – 3.56)	1.32 (0.71 – 2.48)	0.005
CC	5 (2.5)	1 (0.5)	9.70 (1.07 – 87.83)	6.76 (0.63 – 71.88)	0.100
CC+TC	58 (29.0)	32 (16.0)	2.18 (1.32 – 3.61)	1.36 (0.73 – 2.53)	0.002
***CYP2D6***					
GG	86 (43.0	190 (95.0)	1.0	1.0	-
AG	43 (21.5)	6 (3.0)	26.3 (6.54 – 105.5)	42.67 (8.37 – 217.5)	<0.0001
AA	71 (35.5)	4 (2.0)	27.43 (8.73 – 86.14)	20.96 (6.01 – 73.13)	<0.0001
AA+AG	114 (57.0)	10 (5.0)	27.0 (9.95 – 73.24)	27.73 (9.12 – 84.32)	<0.0001
^4^ **Gen- gene interaction between ** ***CYP1A1*** ** and ** ***CYP2D6 *** **(*p* interaction = 0.487 ; SE = 0.225)**					
TT+GG	61 (30.5)	162 (81.0)	1.0	1.0	
CC+TC & GG	25 (12.5)	28 (14.0)	32.68 (10.49 – 101.8)	50.2 (12.57 – 200.7)	
TT & (AA+AG)	81 (40.5)	6 (3.0)	2.02 (0.98 – 4.16)	1.24 (0.48 – 3.22)	
CC+TC & AA+AG	33 (16.5)	4 (2.0)	26.40 (6.19 – 112.6)	8.81 (2.07 – 37.57)	

1 OR=odds ratio.

2 CI=confidence interval

3 Adjusted ORs were obtained from conditional logistic regression models when adjusted for age, family history, parental education level, paternal occupation, place of residence, socioeconomic status and paternal smoking.

High risk of ALL was found in the AA (OR=20.9, P<0.0001) and AG genotypes of CYP2D6*4 (OR=42.6; P<0.0001) and the risk was retained when (AA) and (AG) carriers were grouped together (OR=27.7, P<0.0001). The magnitude of risk associated with AA, AG, and AG + AA was almost similar in the unadjusted model. Further, on analyzing any possible gene–gene interaction, we did not find any significant interactions between *CYP1A1* and *CYP2D6* (p interaction=0.487).

## DISCUSSION

The present study determined the association of *CYP1A1* and *CYP2D6* polymorphisms with ALL risk in Kashmiri population. This population has relative genetic homogeneity [8] which makes it an ideal genetic model for carrying out such studies. We found that unlike CYP1A1*2A, CYP2D6*4 polymorphism is associated with ALL risk. CYP450s are heme-containing enzymes important to phase I-dependent metabolism of drugs and other xenobiotics [15]. Studies have persistently associated polymorphisms in these CYP genes with individual susceptibility to many cancers [16-20]. However, the role of such polymorphism in cancer development is not conclusive [21]. Despite much investigation, little is known about the mechanism of leukemogenesis. Polymorphism in *CYP2D6* gene at position G1934A causes a disruption of the splice site at the intron3/exon4 boundary that leads to incorrect splicing of mRNA resulting in a frame shift and premature termination that generates a truncated protein [22]. These polymorphisms usually lead to no or reduced activity of the CYP2D6 protein, resulting in the poor metabolizer phenotype [23]. Previous studies that have assessed the role of *CYP2D6* genetic variations in susceptibility to ALL have reported mixed results [24, 25]. In the current study, we found that CYP2D6*4 polymorphism is associated with ALL risk in Kashmir. A plausible explanation for our finding could be that as the *CYP2D6* gene is involved in the detoxification of carcinogenic compounds and consequently due to the absence of enzymatic activity genotoxic metabolites gets accumulated in phase I detoxification process resulting in higher risk of ALL [24]. 

*CYP1A1* gene is responsible for metabolic activation of pre-carcinogens [26]. Previous work revealed that the polymorphism of *Msp* I restriction site owing to a T-C variation in the 3’ non-coding region of the CYP1A1 allele is experimentally associated with increased catalytic activity and increase of the amount of DNA adducts in cord blood and placenta of newborns [27]. In the current study, we did not find any association of CYP1A1*2A with susceptibility to develop ALL. However, earlier reports have shown mixed results for this polymorphism and susceptibility to ALL. Studies have either reported the positive association of CYP1A1*2A polymorphism with ALL [14, 25] or no association [28]. This inconsistency in the results obtained in various studies could be attributed to the variable sample size, the heterogeneity of the populations and study design. Epidemiologic studies have propounded that in utero and postnatal exposures to various biological, chemical and physical factors may be important in determining the susceptibility to childhood ALL [29] and as such infants and children may be at greater risk for a variety of environmental toxicants than adults due to their physiologic immaturity and/or differential exposure [27]. Xenobiotics enter the placenta through the maternal circulation [30]. Placenta has the ability to metabolize these compounds through processes similar to those seen in the liver [31]. Therefore, alterations in the placental metabolism could modify the exposure of the developing fetus to harmful electrophiles.

After stratification of data, we found a significant association between paternal smoking with the risk of ALL (P=0.045). Stronger evidence is accumulating now for the role of paternal smoking as reported in several individual studies and meta-analyses of ALL [32, 33]. Whilst smoking clearly impacts DNA damage which is important in carcinogenesis and therefore may influence the risk of ALL [34]. Paternal exposure is of concern, due to possible germline effects for fathers and passive exposure of pregnant women due to cross placental transfer from mother to baby. In utero exposure to low-dose radiation delivered from medical X-rays is one of the few widely recognized risk factors for childhood leukemia [35] and hence the early life exposures to these radiations have been implicated in the etiology of childhood ALL [36]. The increased risk of ALL conferred by in utero X-ray exposure found in this study is in agreement with the recent study [37]. However, other reports did not support these results [38]. To our knowledge, this is the first investigation that attempted to study the impact of polymorphisms of *CYP1A1* and *CYP2D6* on the risk of childhood ALL in Kashmir valley. Besides this study assessed the role of certain non-genetic factors in the development of ALL as well. However further studies with larger sample size are warranted to replicate the findings.
